# Targeted therapies in the management of locally advanced and metastatic pancreatic cancer: a systematic review

**DOI:** 10.18632/oncotarget.25085

**Published:** 2018-04-20

**Authors:** Anjali V. Sheahan, Andrew V. Biankin, Christopher R. Parish, Levon M. Khachigian

**Affiliations:** ^1^ Department of Pathology, Dana-Farber Cancer Institute, Boston 02115, MA, United States of America; ^2^ Wolfson Wohl Cancer Research Centre, University of Glasgow, Glasgow G61 1QH, Scotland, United Kingdom; ^3^ Cancer and Vascular Biology, ACRF Department of Cancer Biology and Therapeutics, The John Curtin School of Medical Research, The Australian National University, Canberra ACT 2601, Australia; ^4^ Vascular Biology and Translational Research, School of Medical Sciences, University of New South Wales, Sydney NSW 2052, Australia

**Keywords:** pancreatic cancer, bibliometric analysis, clinical trials

## Abstract

Pancreatic cancer has a dismal prognosis particularly in patients presenting with unresectable tumors. We performed a bibliometric analysis of clinical trials for pancreatic cancer conducted between 2014-2016 focusing on patients that presented with unresectable (locally advanced or metastatic) tumors. We discuss a range of studies that employed FOLFIRINOX, the gemcitabine + nab-paclitaxel combination and studies that used molecularly-targeted therapy. Major areas of focus have been dual targeting of EGFR and VEGFR, immunotherapy or a multimodal approach – combining chemotherapy with radiotherapy. We also point out the need for molecular selection for low prevalence subtypes. Key insights sourced from these pivotal trials should improve clinical outcomes for this devastating cancer.

## INTRODUCTION

The American Cancer Society reports increasing death rates for people who develop cancer of the pancreas, with a 5-year survival rate of only 8% [1]. Most recently global data, acquired as part of the GLOBOCAN project in 2012, revealed pancreatic cancer to be the 7^th^ most common cause of cancer death, accounting for approximately 338000 cancer diagnoses and 331000 deaths. In Western societies, it is the 4^th^ leading cause of cancer death, and recently overtook breast cancer to become the 3^rd^ leading cause in the USA, and is projected to be the second leading cause within a decade [2]. These abysmal statistics are partly attributed to the fact that 53% of pancreatic cancers are diagnosed metastatic and 28% with loco-regional disease, with survival rates of 2% and 11%, respectively. As resection is the only curative therapy for pancreatic cancer patients, management is mainly confined to chemotherapy, which is sometimes combined with radiotherapy in loco-regional disease. Since our last review of therapeutic strategies for the treatment of pancreatic cancer [3], major advances have been made in therapies available to patients with locally advanced pancreatic cancer (LAPC) and metastatic pancreatic cancer (MPC). Here we examined the changing landscape of the clinical management of unresectable pancreatic adenocarcinomas (PDAC). Bibliometric analysis was also utilized to gauge the level of scientific interest in particular therapeutic strategies.

## METHODS

**Figure 1:**
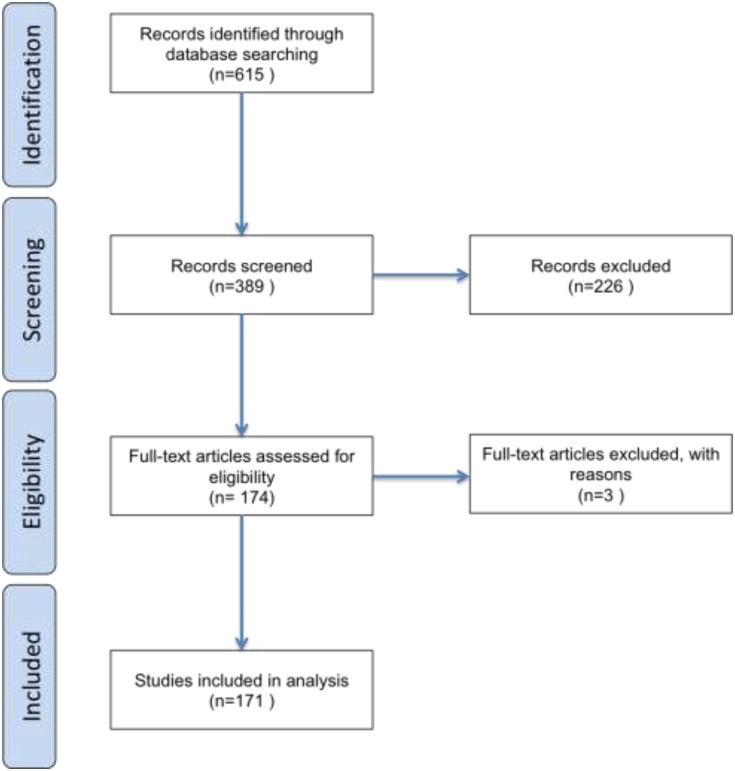
Flow chart depicting how bibliometric information was processed in the course of this review

For bibliometric analysis the PubMed database was queried using the “trial AND (pancreas OR pancreatic) AND (cancer OR tumor OR adenocarcinoma) AND (unresectable OR advanced OR metastatic)” search term, specifying publication dates from 2014/01/01 to 2016/12/31 to identify relevant articles published since 2014. Figure 1 provides a flowchart showing how bibliometric information was processed during the course of systematic review. Of the 615 identified articles, reviews, case-studies, sub-group analyses and meta-analyses were excluded. Reports that did not explore therapeutic strategies for the purpose of treating unresectable pancreatic ductal adenocarcinomas, i.e. ultimately increasing overall survival (OS) of patients, were also excluded, leaving 171 relevant articles. This included first-line and second-line studies, and dose-finding studies performed in patients presenting with a broad grouping of advanced solid tumors as long as pancreatic cancer patients were included. Studies that primarily investigated prophylactic treatments were excluded. Phase I trials investigating therapeutics in multiple advanced solid tumor types were included in this systematic review but survival data was only used if pancreatic cancer data was reported. Citation data was retrieved from Web of Science.

## STANDARD OF CARE

New standards of care have been established from two landmark phase III trials that demonstrated significant improvements in OS in patients that presented with metastatic pancreatic cancer. An overview of the clinical trials that have influenced this standard of care, outlining OS achieved using different drug combinations, is included in Table 1. In 2011, Conroy *et al.* (PRODIGE) [4] demonstrated that an OS of 11.1 months was achievable in chemotherapy-naïve patients treated with a combination of 5-fluorouracil, leucovorin, irinotecan and oxaliplatin (FOLFIRINOX), compared to 6.8 months in patients treated with gemcitabine alone. Subsequently, Von Hoff *et al.* (MPACT) [5], also achieved a significant improvement in OS in patients treated with gemcitabine and the stromal-targeting nanoparticle albumin-bound paclitaxel (nab-paclitaxel) (GNP) when compared with patients receiving gemcitabine monotherapy (8.5 vs 6.7 months).

**Table 1:** Studies that influenced current standard of care, displayed in order of publication

OS	Title	Reference	Patients	Phase	Randomization
5.65 months Gem, 4.41 months 5-fluorouracil	Improvements in survival and clinical benefit with gemcitabine as first-line therapy for patients with advanced pancreas cancer: a randomized trial.	Burris et al. J Clin Oncol. 1997	126	III	Yes
6.24 months Gem + Erlotinib, 5.91 months Gem	Erlotinib plus gemcitabine compared with gemcitabine alone in patients with advanced pancreatic cancer: a phase III trial of the National Cancer Institute of Canada Clinical Trials Group.	Moore et al. J Clin Oncol. 2007	569	III	Yes
11.1 months FOLFIRINOX, 6.8 months Gem	FOLFIRINOX versus gemcitabine for metastatic pancreatic cancer.	Conroy et al. N Engl J Med. 2011	342	III	Yes
8.5 months Gem + nab-paclitaxel, 6.7 months Gem	Increased survival in pancreatic cancer with nab-paclitaxel plus gemcitabine.	Von Hoff et al. N Engl J Med. 2013	861	III	Yes
6.1 months nano-irinotecan + 5-fluorouracil + folinic acid, 4.2 months 5-fluorouracil + folinic acid	Nanoliposomal irinotecan with fluorouracil and folinic acid in metastatic pancreatic cancer after previous gemcitabine-based therapy (NAPOLI-1): a global, randomised, open-label, phase 3 trial	Wang-Gillam et al. Lancet 2016 (Epub 2015)	417	III	Yes

Median overall survival (OS) in advanced pancreatic cancer patients is significantly improved using the gemcitabine + nab-paclitaxel or FOLFIRINOX combinations when compared to the previous standard of care, gemcitabine (Gem). The addition of the targeted therapy, erlotinib, to conventional gemcitabine treatment had been shown to have a small but significant effect on OS in an earlier report. The combination of nanoliposomal irinotecan with 5-fluouracil and folinic acid has been FDA approved in the 2^nd^ line setting.

**Figure 2:**
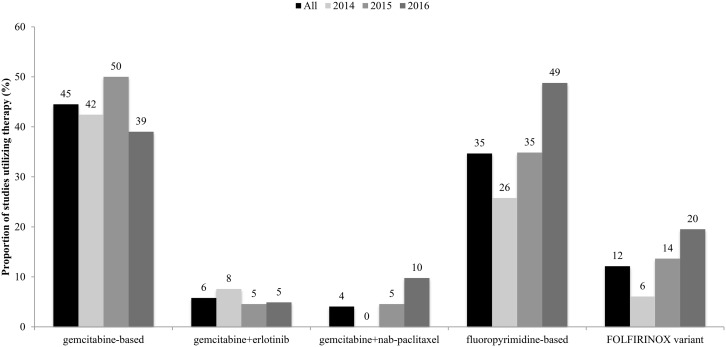
Proportion of studies in 2014-2016 that employed conventional chemotherapies Gemcitabine- and fluoropyrimidine-based therapies were highly utilized in clinical trials, with increases seen in the employment of the latter over these 3 years. There were also increases in the utilization of the FOLFIRINOX and gemcitabine+nab-paclitaxel combinations in the same time period.

Increased interest and adoption of the FOLFIRINOX combination and its variants, and the GNP combination are reflected in increases in the number of clinical trials that have employed these strategies over the last 3 years (Figure 2). In fact, there was an increase in the use of fluoropyrimidine-based therapies, which includes 5-fluorouracil, or its prodrugs capecitabine or tegafur in this same period. Gemcitabine-based therapies, including CO-101 – a lipid drug conjugate of gemcitabine – remained highly utilized in the treatment of PDAC. However, there was a decrease in the proportion of trials that employed gemcitabine-based therapies from 2015 to 2016. There was also little change in the utilization of the gemcitabine/erlotinib combination, which although shown to significantly extend OS when compared to gemcitabine (6.24 months vs 5.91 months) [6], provided only a modest survival benefit.

While FOLFIRINOX may, on the surface, appear to outperform the GNP combination, we note that PRODIGE was a one country study with narrow scope, whereas MPACT was an international study that included older patients [median: 61 (range: 25-76) vs median: 63 (range: 27-88)] and those with a poorer performance status [1 patient (0.3%) with ECOG performance status > 1 vs 65 patients (7.6%) with Karnofsky performance status <80]. Both strategies were associated with adverse events including grade 3 or higher neutropenia (45.7% vs 38%) and fatigue (23.6% vs 17%). Use of modified FOLFIRINOX, where irinotecan and bolus 5-fluorouracil is reduced by 25%, resulted in a significant reduction of grade 3 or higher neutropenia (12.2%) and fatigue (12.2%) while achieving an OS of 10.2 months in patients with metastatic PDAC and 26.6 months in patients with LAPC [7]. This Phase II study was also conducted in a younger population [median: 62 (range: 50-77)] with ECOG performance status scores <2. A meta-analysis conducted by Suker et al. [8] however, recently demonstrated the value of FOLFIRINOX in the treatment of LAPC with a median OS of 24.2 months in the pooled patient population. Proportion of patients that underwent surgery after FOLFIRINOX was 25.9% and R0 resection was achieved in 74% of these patients.

The GNP combination has also shown promise in the second-line setting in MPC patients refractory to FOLFIRINOX therapy [9], resulting in an OS of 8.8 months or 18 months since the start of first-line therapy. This study included 12 patients (21%) with an ECOG performance status of 2, and 40% of patients reported grade 3 or higher adverse events, including neurotoxicity (12.5%) and neutropenia (12.5%).

Advancements that have followed from the success of the FOLFIRINOX combination include the development of nanoliposomal irinotecan (MM-398), designed to extend duration of the drug in the circulation, and preferential activation in the tumor. The highly cited findings of the NAPOLI-1 trial, which assessed nanoliposomal irinotecan in combination with 5-FU and folinic acid in MPC patients after failure of gemcitabine-based therapy, achieved a significant improvement in OS compared to patients that received only 5-FU and folinic acid (6.1 months vs 4.2 months) [10]. As a result, this regimen became the first FDA approved second-line therapy (after gemcitabine failure) in patients that present with MPC. A Phase II trial investigating nanoliposomal irinotecan-based combinations in the first-line setting is underway [11].

It is important to note that US treatment guidelines recommend that patients that present with unresectable pancreatic cancer, particularly those with a good performance status, receive treatment within a clinical trial [12]. However, a 2013 study reported that only 4.57% of pancreatic cancer patients enroll into clinical trials [13] - a statistic that could be improved with greater awareness of pancreatic cancer trials and improved discussion between patients and caregivers [14].

In summary, all studies have shown incremental benefit only, with the choice between the 2 standards of care currently available (FOLFIRINOX and Gemcitabine + nab-Paclitaxel) only based on performance status as no predictive patient selection tools currently exist.

## MOLECULARLY-TARGETED THERAPY

Pancreatic tumors tend to be heterogeneous, presenting a challenge for treatment [9, 15, 16]. Molecularly-targeted therapies offer physicians the opportunity to tailor a strategy to the unique properties of a patient's individual tumor. Further by selecting targets restricted to cancer or cancer-associated cells, and not healthy cells, these therapies potentially minimize side effects associated with conventional chemotherapeutic strategies that employ cytotoxic agents.

**Figure 3:**
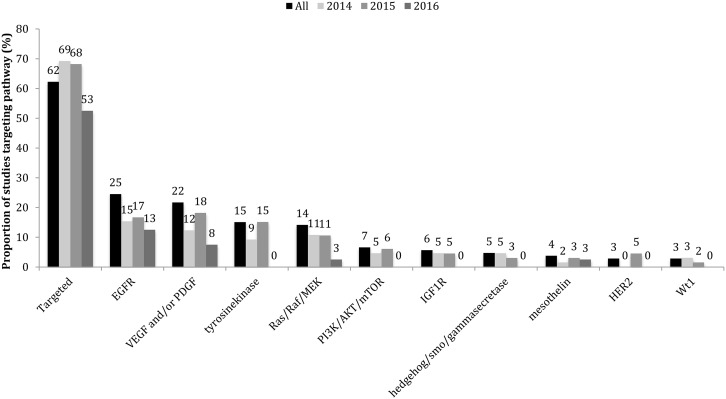
Proportion of studies in 2014-2016 that employed molecularly-targeted chemotherapies Data is displayed in the order of the popularity of the target (the proportion of studies that utilized an inhibitor of that target). Molecularly targeted therapies were used in the majority of clinical trials investigating therapeutics in advanced pancreatic cancer patients in this timeframe.

A molecularly-targeted therapy was explored in 97 (54%) of the 178 clinical studies that investigated therapeutic strategies in patients presenting with unresectable pancreatic cancer (Figure 3). When dose-finding studies were excluded, 63 studies (49%) of the remaining 129 studies employed molecularly-targeted therapy. Many of the most cited reports published in 2014–2016 (Tables 2–4) investigated a molecularly-targeted therapy.

**Table 2:** Top cited articles published in 2014, 2015, and 2016 ranked by the number of citations (online publication or e-publication date used as date of publication)

Citations	Title	Reference	Patients	Phase	Randomization
116	Second-line oxaliplatin, folinic acid, and fluorouracil versus folinic acid and fluorouracil alone for gemcitabine-refractory pancreatic cancer: outcomes from the CONKO-003 trial	Oettle et al. J Clin Oncol. 2014	168	III	Yes
79	Gemcitabine and capecitabine with or without telomerase peptide vaccine GV1001 in patients with locally advanced or metastatic pancreatic cancer (TeloVac): an open-label, randomised, phase 3 trial	Middleton et al. Lancet Oncol. 2014	1062	III	Yes
71	Phase 2 multi-institutional trial evaluating gemcitabine and stereotactic body radiotherapy for patients with locally advanced unresectable pancreatic adenocarcinoma	Herman et al. Cancer. 2015	49	II	No
138	Safety and survival with GVAX pancreas prime and Listeria Monocytogenes-expressing mesothelin (CRS-207) boost vaccines for metastatic pancreatic cancer	Le et al. J Clin Oncol 2015	90	II	Yes
114	Nanoliposomal irinotecan with fluorouracil and folinic acid in metastatic pancreatic cancer after previous gemcitabine-based therapy (NAPOLI-1): a global, randomised, open-label, phase 3 trial	Wang-Gillam et al. Lancet 2016 (Epub 2015)	417	III	Yes
81	Metformin in patients with advanced pancreatic cancer: a double-blind, randomised, placebo-controlled phase 2 trial	Kordes et al. Lancet Oncol 2015	121	II	Yes
77	Effect of Chemoradiotherapy vs Chemotherapy on Survival in Patients With Locally Advanced Pancreatic Cancer Controlled After 4 Months of Gemcitabine With or Without Erlotinib: The LAP07 Randomized Clinical Trial	Hammel et al. JAMA 2016	449	III	Yes
46	Targeting tumour-associated macrophages with CCR2 inhibition in combination with FOLFIRINOX in patients with borderline resectable and locally advanced pancreatic cancer: a single-centre, open-label, dose-finding, non-randomised, phase 1b trial	Nywening et al. Lancet Oncol 2016	47	Ib	No
18	Final analysis of a phase II study of modified FOLFIRINOX in locally advanced and metastatic pancreatic cancer	Stein et al. Br J Cancer 2016	75	II	No

**Table 3:** Top 8 articles investigating therapies in patients that present with metastatic pancreatic cancer (MPC), ranked according to overall survival (OS)

OS	Title	Reference	Patients	Phase	Randomization
717 days (strong DTH reactions)	Treatment with chemotherapy and dendritic cells pulsed with multiple Wilms’ tumor 1 (WT1)-specific MHC class I/II-restricted epitopes for pancreatic cancer	Koido et al. Clin Cancer Res. 2014	10	I	No
18 months, 8.1 months after first-line therapy	Nab-paclitaxel plus gemcitabine for metastatic pancreatic adenocarcinoma after Folfirinox failure: an AGEO prospective multicentre cohort	Portal et al. Br J Cancer 2015	57	II	No
13.5 months	Phase I/II study of nab-paclitaxel plus gemcitabine for chemotherapy-naive Japanese patients with metastatic pancreatic cancer	Ueno et al. Cancer Chemother Pharmacol 2016	34	I/II	No
13.5 months fluoro after GNP, 9.5 months fluoro after Gem	Second-line therapy after nab-paclitaxel plus gemcitabine or after gemcitabine for patients with metastatic pancreatic cancer	Chiorean et al. Br J Cancer 2016	347	retrospective	
11.9 months GEMOXEL, 7.1 months Gem	Gemcitabine, oxaliplatin, and capecitabine (GEMOXEL) compared with gemcitabine alone in metastatic pancreatic cancer: a randomized phase II study	Petrioli et al. Cancer Chemother Pharmacol 2015	67	II	Yes
11 months FIRGEM, 8.2 months Gem	Fixed-dose rate gemcitabine alone or alternating with FOLFIRI3 (irinotecan, leucovorin and fluorouracil) in the first-line treatment of patients with metastatic pancreatic adenocarcinoma: an AGEO randomised phase II study (FIRGEM)	Trouilloud et al. Eur J Cancer 2014	98	II	Yes

Gem indicates gemcitabine, fluoro indicates fluoropyrimidine-based therapy, GNP indicates gemcitabine+nab-paclitaxel, DTH indicates delayed-type hypersensitivity.

**Table 4:** Top 8 articles investigating therapies in patients that present with locally advanced pancreatic cancer (LAPC), ranked according to median overall survival (OS) achieved

OS	Title	Reference	Patients	Phase	Randomization	R0 resection
29 months (arterial involvement), 42+ months (venous involvement)	Neoadjuvant gemcitabine, docetaxel, and capecitabine followed by gemcitabine and capecitabine/radiation therapy and surgery in locally advanced, unresectable pancreatic adenocarcinoma	Sherman et al. Cancer 2015	45	III	No	67% (arterial involvement), 73% (venous involvement)
26.6 months in LAPC	Final analysis of a phase II study of modified FOLFIRINOX in locally advanced and metastatic pancreatic cancer	Stein et al. Br J Cancer 2016	75	II	No	41.9%
12.3 months in all treated patients, 19.5 months with a dose of 1 mg/kg	Phase I Clinical Trial to Determine the Feasibility and Maximum Tolerated Dose of Panitumumab to Standard Gemcitabine-Based Chemoradiation in Locally Advanced Pancreatic Cancer	Van Zweeden et al. Clin Cancer Res 2015	14	I	No	Not determined
18.8 months	Baseline metabolic tumor volume and total lesion glycolysis are associated with survival outcomes in patients with locally advanced pancreatic cancer receiving stereotactic body radiation therapy	Dholakia et al. Int J Radiat Oncol Biol Phys 2014	32	II	No	Not determined
18.4 months LAPC, 14.4 months BRPC	The Role of Stereotactic Body Radiation Therapy for Pancreatic Cancer: A Single-Institution Experience.	Moningi et al. Ann Surg Oncol. 2015	88 (74 LAPC)		No	84% margin negative, 16% pathologic complete response

CRT indicates chemoradiotherapy, IC indicates induction chemotherapy, and BRPC indicates borderline resectable pancreatic cancer. Rates of achieving microscopic tumor clearance (R0) with resections are also displayed.

Epidermal growth factor receptor (EGFR) or HER1, which resides on the surface of the cancer cell, remained the most utilized target, accounting for 27 (15%) of the 178 trials in our review. The specific EGFR inhibitors, erlotinib, and cetuximab, accounted for 16 and 7 of these trials, respectively. A Phase II trial where LAPC patients that responded to gemcitabine/capecitabine therapy were randomized to receive radiotherapy, and a fluoropyrimidine-based therapy (capecitabine or uracil/tegafur plus leucovorin), reported a non-significant increase in the OS achieved in patients that also received cetuximab (22 months vs 15.8 months) [17]. Further, patients with low baseline circulating miR-21 had an OS of 15.3 months, compared to 5.1 months in patients with high miR-21 levels in plasma (also not significant). Low enrolment numbers (17 patients, 13 analyzed) may account for a lack of statistical power.

Other EGFR-targeted therapeutics (panitimumab, afatinib, lapatinib and vandetanib) and the HER2 inhibitor, trastuzumab, were each used in just one study. A Phase I dose finding study that added the EGFR inhibitor panitimumab to gemcitabine-based chemoradiation in LAPC patients, appears promising, reporting an OS of 12.3 months (19.5, 17 and 9.1 months at dose rates of 1, 1.5 and 2 mg/kg, respectively) [18]. Combined inhibition of EGFR and HER2 using cetuximab and trastuzumab, in patients with gemcitabine-refractory MPC, resulted in an OS of 4.6 months [19]. Another Phase II trial [20] employing EGFR/HER2 tyrosine kinase inhibitor (TKI), lapatinib, in combination with capecitabine as second-line therapy in patients with gemcitabine-refractory MPC reported an OS of 5.2 months. However, the discrepancy in OS achieved in patients that responded with stable disease (8.3 months) vs progressive disease (2.9 months) suggests tumor-specific responses that may benefit from biomarker-based patient stratification.

Twenty four studies (13%) employed an inhibitor of vascular endothelial growth factor (VEGF) or platelet-derived growth factor (PDGF), including 14 studies that employed a tyrosine kinase inhibitor (TKI). The VEGFA inhibitor bevacizumab was the most utilized inhibitor of angiogenesis pathways, and was employed in 6 studies (3%), and was used in combination with an EGFR inhibitor (erlotinib or cetuximab) in 4 of these studies. In fact, the addition of the bevacizumab/cetuximab combination improved clinical outcomes in patients presenting with LAPC and MPC (OS: 13 months) when compared with patients receiving conventional chemotherapy (OS: 7 months) [21]. The addition of the targeted bevacizumab/erlotinib combination to the gemcitabine/capecitabine chemotherapy combination achieved an OS of 12.6 months (10.1 months in MPC patients) [22]. Further, an OS of 17.4 months was achieved in a trial where LAPC patients were treated with capecitabine, erlotinib, and bevacizumab with concurrent radiotherapy [23].

TKIs were employed in 17 (10%) studies included in this review. Sorafenib, a TKI of VEGFR, PDGFR and Raf, was utilized in 5 studies (3%). One of these studies [24], a Phase I trial treating LAPC patients with a combination of gemcitabine and sorafenib, with concurrent radiation therapy, achieved an OS of 12.6 months. Another Phase I trial [25] investigating the gemcitabine/sorafenib combination in patients that presented with LAPC and MPC, linked serum levels of an indirect marker of angiogenesis (lactate dehydrogenase) with response to chemotherapy containing sorafenib.

The Ras/Raf/MEK pathway was targeted in 13 (7%) of trials included in this review. A randomized Phase II trial evaluating gemcitabine combined with trametinib, an oral MEK inhibitor in patients that presented with MPC showed no statistically significant improvement in OS compared to the gemcitabine + placebo control (8.4 months vs 6.7 months) [26]. A Phase II/III trial of a combination of gemcitabine with rigosertib, a Ras mimetic and small molecule inhibitor of PLK1 and PI3K, also showed no significant survival benefit when compared with gemcitabine in patients that present with MPC (6.1 months vs 6.4 months) [27]. A Phase II trial of the MEK1/2 inhibitor, selumetinib, delivered in combination with erlotinib, in the second-line setting, achieved an OS of 7.3 months [28].

Studies that targeted the PI3K/AKT/mTOR pathway accounted for 7 (4%) of trials included in this review. Of these, the most significant was a Phase II study of the oral mTOR inhibitor, everolimus, in combination with capecitabine achieved an OS of 8.9 months in patients that presented with advanced pancreatic cancer (94% MPC) [29].

Six (3%) studies employed an IGF1R inhibitor. A Phase III trial of ganitumab, a monoclonal antibody against IGF1R, delivered in combination with gemcitabine, failed to demonstrate a survival benefit in MPC patients when compared to gemcitabine alone [7.0 months (gemcitabine + 12 mg/kg ganitumab), 7.1 months (gemcitabine + 20 mg/kg ganitumab), 7.2 months (gemcitabine + placebo)] [30]. A Phase I study of somastatin analog and IGF1R inhibitor, pasireotide, achieved an OS of 6.9 months in LAPC and MPC patients [31]. Dual targeting of EGFR and IGF1R using erlotinib and cixutumumab did not enhance the efficacy of gemcitabine anymore than erlotinib alone [6.7 months (gemcitabine + erlotinib + cixutumumab) vs 7.0 months (gemcitabine + erlotinib)] [32].

Hedgehog signaling elements (hedgehog/smo/γ secretase) accounted for 5 (3%) of studies included in our dataset. Interest in this signaling pathway stems from its roles in pancreatic cancer stem cell maintenance and, as it is expressed by stromal cells, tumor hypoxia. However, clinical trials that targeted hedgehog elements failed to demonstrate survival benefits [33–35].

Our analysis indicated a slight decline in the percentage of clinical trials that employed targeted therapies from 2014 to 2015, and again from 2015 to 2016. Speculatively, this may be indicative of increased interest in improving FOLFIRINOX- and GNP-based combinations, stemming from the recent successes of the MPACT and PRODIGE clinical trials. As we only examined 3 years of trials reported in PubMed, and that too in the immediate aftermath of the MPACT trial, it will be interesting to follow the direction of these clinical studies, particularly in terms of the preferred future use of molecular selection.

In summary, despite many studies, the only targeted therapy with evidence of efficacy in late phase studies is erlotinib, yet the effect is so small that it has not been broadly adopted in routine practice. Molecularly-targeted therapies tested in unselected patients with pancreatic cancer may be effective in small subgroups, but the inability to predict these prior to treatment precludes their use currently.

## STROMAL TARGETS

Tumor hypoxia, a consequence of hypoperfusion and desmoplasia that have become characteristic features of PDAC, has long been under intense scrutiny, particularly for approaches that improve drug efficacy by overcoming resistance to treatment. In fact, drugs that target hypoxia are of interest in therapeutic approaches that target the dense, poorly vascularized stroma that encapsulates PDAC tumor cells. The success of nab-paclitaxel, which is thought to target SPARC (secreted protein-acid rich in cysteine), that is expressed by stromal cells, further encouraged this field of study.

However, the combination of gemcitabine and the hypoxia-activated pro-drug, TH-302, did not significantly improve OS in patients that presented with advanced pancreatic cancer [9.2 months (340 mg/m^2^), 8.7 months (240 mg/m^2^) vs 6.9 months (gemcitabine alone)] [36]. This highly cited article reported significant improvement in PFS [5.6 months (pooled combination arms) vs 3.6 months (gemcitabine alone)]. A randomized Phase III trial also showed no significant difference in OS (8.7 months vs 7.6 months) and significant differences in PFS (5.5 months vs 3.7 months) [37]. A Phase I dose escalation trial evaluating TH-302 in combination with GNP was terminated early as development of this drug was abandoned by the sponsoring company [38].

## IMMUNOTHERAPY

Twenty (11%) of the clinical studies employed an immunotherapy, which seems to be a particularly promising avenue of study in patients that present with MPC (Table 3). A particularly interesting Phase Ib trial investigated an oral CCR-2 inhibitor (PF-04136309), a strategy aimed to target CCL2-CCR2 chemokine signaling that mediates tumor-associated macrophage recruitment, and therefore restores anti-tumor immunity [39]. Delivered in combination with FOLFIRINOX, this inhibitor achieved objective tumor responses in 16% of patients and local tumor control in 97% of patients.

A Phase I trial [40] investigating the clinical response of MPC patients to a combination of gemcitabine and mature dendritic cells pulsed with MHC class I/II-restricted Wilms’ tumor 1 (Wt1) peptides, showed improvements in OS in patients that displayed WT1-specific delayed-type hypersensitivity (DTH) (717 days). An earlier study [41] of a Wt1-based cancer vaccine delivered in combination with gemcitabine therapy had reported an OS of 10.9 months in DTH positive LAPC/MPC patients, compared to 3.9 months in DTH negative patients. Another Japanese Phase I [42] study of the same therapy in LAPC and MPC patients reported an OS of 243 days.

As with other cancer types, there has been considerable interest in the development and validation of cancer vaccines. An investigation of a mutant Ras peptide vaccine delivered in combination with interleukin-2 (Arm 1) or granulocyte-macrophase colony-stimulating factor (GM-CSF) (Arm 2) or both (Arm 3) to enhance the vaccine immune response, achieved an OS of 16.6 months in 53 advanced cancer patients (including 11 pancreatic cancer patients) with no significant difference between arms [43].

GVAX, GM-CSF-secreting whole allogeneic pancreatic cancer cells, is often delivered in combination with cyclophosphamide (Cy), which inhibits regulatory T-cells. The addition of *Listeria monocytogenes* organisms expressing mesothelin (CRS-207), another cancer vaccine that induces innate and adaptive immunity, to Cy/GVAX extended OS from 3.9 months to 6.1 months [44]. The majority of patients (97%) had received prior chemotherapy and 51% had received more than two regimens. In a subsequent Phase IIb trial, conducted in patients that had failed at least two prior therapies in the metastatic setting, this combination failed to meet the primary endpoint of an improvement in OS [3.8 months (GVAX/Cy/CRS-207) vs 5.4 months (CRS-207 alone) vs 4.6 months (chemotherapy)] [45].

Another vaccine of interest is GV1001, made up of telomerase peptides, designed to train the immune system to recognize this common cell surface protein. GM-CSF is commonly administered to patients prior to GV1001 therapy to boost effectiveness. A Phase III trial to assess GV1001 in combination with gemcitabine and capecitabine, failed to significantly improve overall survival in LAPC or MPC patients [46]. Patients received the gemcitabine/capecitabine combination with sequential GM-CSF/GV1001 (OS: 6.9 months), concurrent GM-CSF/GV1001 (OS: 8.4 months), or alone (OS: 7.9 months).

In summary, although most single agent immunotherapeutic strategies in pancreatic cancer have been disappointing, novel combinations, based on preclinical evidence of efficacy [47] are being explored.

## RADIOTHERAPY

Radiotherapy was utilized in 32 studies (18%), 30 (17%) of which also employed a chemotherapy. Twenty (11%) studies that employed a radiotherapy also employed a gemcitabine-based therapy, 20 (11%) employed a fluoropyrimidine-based therapy and 11 (6%) employed capecitabine. In the highly cited LAP07 clinical trial [48], 442 LAPC patients were first randomized to receive 4 months of induction chemotherapy with gemcitabine alone or the gemcitabine/erlotinib combination. The 269 patients that showed progression-free disease at 4 months were then randomized to receive 2 more months of the same chemotherapy with or without the addition of capecitabine-based radiotherapy. There was no significant difference in the OS achieved in patients receiving chemotherapy (16.5 months) vs chemoradiation (15.2 months) and no significant difference between patients receiving the gemcitabine/erlotinib combination (11.9 months) when compared to patients receiving gemcitabine monotherapy (13.6 months).

The case for pursuing multimodal therapeutic strategies in pancreatic cancer is particularly supported by the fact that 7 of the 8 studies that reported the highest OS in LAPC patients employed some form of radiotherapy (Table 4), with the hope that this would result in a downgrading of the tumor, enabling curative resection.

Six studies (3%) explored stereotactic body radiation therapy (SBRT). SBRT involves precise mapping of the tumor using imaging strategies, enabling high dose radiotherapy to be delivered directly to the tumor, typically at multiple radiation beam angles. This ablative strategy is attractive as biologically potent doses of radiation can be delivered to a localized tumor while sparing healthy tissue, hopefully resulting in greater efficacy with fewer side effects. All 6 articles that utilized SBRT did so in the LAPC setting. Survival was measured in 5 of these studies, and an OS of 18+ months was achieved in 4 (2%) studies with low toxicities [49–52]. Again, a study that compared SBRT (OS: 18.8 months) to traditional chemoradiation (OS: 13.6 months) found the difference in OS to be not significant [52], which was attributed by the authors to a small patient population. Four studies that employed a chemotherapeutic, used a gemcitabine-based therapeutic. The mFOLFIRINOX/SBRT multimodality combination is currently under investigation in a Phase III trial at Stanford University [53]. It will be interesting to see how further refinements to SBRT-based chemoradiation impact survival outcomes.

In summary, similar to other treatment options, radiotherapy is likely effective in subgroups of patients that are currently not predictable ahead of treatment.

## ALTERNATIVE MODALITIES

Clinical trials that have evaluated alternative therapeutic modalities have been promising (Table 5). One Phase I/II study evaluated a second-generation photosensitizer (verteporfin) combined with photodynamic therapy (PDT) in 15 patients that presented with LAPC [54]. While patients included in this study received a range of oncological treatments before or after PDT, an OS of 8.8 months (15.5 months after diagnosis) was reached. An OS of 17.6 months was achieved in a small cohort of LAPC and MPC patients (n=10), treated with a combination of gemcitabine and microbubbles under sonication [55]. The combination of ultrasound and microbubbles appeared to enhance the efficacy of conventional chemotherapy in this Phase I trial without increasing frequency of adverse effects.

**Table 5:** Other therapies that achieved high median overall survival (OS) in patients that presented with advanced pancreatic cancer, ranked according to OS

OS	Title	Reference	Patients	Phase	Randomization
17.6 months	A human clinical trial using ultrasound and microbubbles to enhance gemcitabine treatment of inoperable pancreatic cancer	Dimcevski et al. J Control Release 2016	10	I	No
16.6 months	A phase I trial of gemcitabine, S-1 and LV combination (GSL) therapy in advanced pancreatic cancer	Nakai et al. Cancer Chemother Pharmacol 2014	15	I	No
13.4 months	Phase I study assessing the feasibility of the triple combination chemotherapy of SOXIRI (S-1/oxaliplatin/irinotecan) in patients with unresectable pancreatic ductal adenocarcinoma	Yanagimoto et al. Cancer Chemother Pharmacol 2016	15	I	No
13 months (conventional + targeted), 7 months (conventional)	Combination of Two Targeted Medications (Bevacizumab Plus Cetuximab) Improve the Therapeutic Response of Pancreatic Carcinoma	Tai et al. Medicine (Baltimore) 2016	59	retrospective	
12.7 months (S, LDH<=UNR), 5.9 months (S, LDH>UNR), 8.6 months (no S, LDH<=UNR, 5.2 months (no S, LDH>UNR)	The value of lactate dehydrogenase serum levels as a prognostic and predictive factor for advanced pancreatic cancer patients receiving sorafenib	Faloppi et al. Oncotarget 2015	71	retrospective	
12.6 months first line	First-in-man phase 1 clinical trial of gene therapy for advanced pancreatic cancer: safety, biodistribution, and preliminary clinical findings	Buscail et al. Mol Ther 2015	22	I	No
12.6 months all patients, 10.1 months MPC	The combination of a chemotherapy doublet (gemcitabine and capecitabine) with a biological doublet (bevacizumab and erlotinib) in patients with advanced pancreatic adenocarcinoma The results of a phase I/II study	Watkins et al. Eur J Cancer 2014	44	I/II	No
8.9 months all patients, 12.4 months (1st line), 5.9 months (2nd line)	Phase II study of capecitabine and the oral mTOR inhibitor everolimus in patients with advanced pancreatic cancer	Kordes et al. Cancer Chemother Pharmacol 2015	31	II	No

MPC indicates metastatic pancreatic cancer, LDH indicates lactate dehydrogenase, UNR indicates upper normal rate.

## CONCLUDING REMARKS

While recent activity and advancements in the treatment options available to pancreatic cancer patients has been encouraging, it is readily evident that we are far from a cure. In particular, in patients that present with MPC, it is disheartening to see the attention that has been given to combinations that extend survival at the expense of quality of life. Rationally selected molecularly-targeted therapies have the potential for efficacious therapy with a low probability for side effects, but as PDAC is a molecularly heterogeneous disease, one would expect heterogeneous responses. Combinations of therapies that targeted EGFR and VEGFR appear to have resulted in increases in OS in clinical trials, but leave room for improvement. Concurrent inhibition of critical nodes appears to be a rational approach in achieving synergistic efficacy, but has the potential for synergistic toxicities. The clinical benefits of personalized medicine, where patients receive therapy based on their tumor's molecular analysis, is currently under investigation [56, 57]. The feasibility of using genomic profiling to identify actionable molecular targets to inform the treatment of pancreatic cancer patients has been recently demonstrated [58]. In the context of molecularly-targeted therapy, it is likely that responses will be dictated by the tumor's molecular signature. Therefore, the performance of molecularly-targeted therapy in clinical trials may be improved by employing molecular selection of patients. Post-hoc selection of MPC patients with low hENT1 failed to demonstrate the superiority of CO-101 over gemcitabine in this population, even though the former was rationally designed to be delivered to cells independently of hENT1 [59]. Hyaluronan A is being used to select patients for a Phase III trial testing PEGylated recombinant human hyaluronidase in combination with GNP compared to GNP alone [60]. MPC or LAPC patients enrolling in a Phase II trial of gemcitabine, cisplatin with or without a PARP inhibitor, veliparib, or veliparib alone are required to provide confirmation of a BRCA1, BRCA2 or PALB2 mutation [61]. PARP inhibitors, rucaparib and olaparib, are also under clinical investigation in patients that present with BRCA/PALB-associated MPC [62, 63]. Successes in such trials may pave the way for rational molecular screening of patients enrolling in clinical studies, but careful post-hoc analysis of pancreatic tumors will greatly advance our knowledge of underlying genetic markers that influence patient responses. This will hopefully result in more successful clinical trials and approvals based on molecularly-based disease indications.

The landscape of pancreatic cancer therapy has moved forward in the last 4-5 years, with shifts in the standard of care, and advances in our understanding of the molecular basis of the disease, which has been greatly informed by genome sequencing. Greater clinical trial participation, rational design of trials, which may include molecular screening of patients, and pursuit of novel therapeutic strategies should result in more powerful therapeutic options for this devastating disease.
